# Amadori-glycated phosphatidylethanolamine enhances the physical stability and selective targeting ability of liposomes

**DOI:** 10.1098/rsos.171249

**Published:** 2018-02-14

**Authors:** Taiki Miyazawa, Reina Kamiyoshihara, Naoki Shimizu, Takahiro Harigae, Yurika Otoki, Junya Ito, Shunji Kato, Teruo Miyazawa, Kiyotaka Nakagawa

**Affiliations:** 1 Food and Biodynamic Chemistry Laboratory, Graduate School of Agricultural Science, Tohoku University, 468-1 Aramaki Aza Aoba, Aoba-ku, Sendai, Miyagi, 980-0845, Japan; 2 Department of Cell Biology, Division of Host Defense Mechanism, Tokai University School of Medicine, Isehara, Kanagawa 259-1193, Japan; 3 Food and Biotechnology Innovation Project, New Industry Creation Hatchery Center (NICHe), Tohoku University, Sendai 980-8579, Japan; 4 Food and Health Science Research Unit, Graduate School of Agricultural Science, Tohoku University, Sendai 980-0845, Japan

**Keywords:** Amadori-glycated phosphatidylethanolamine, curcumin, glucose transporter, glycolipid, liposome, Maillard reaction product

## Abstract

Liposomes consisting of 100% phosphatidylcholine exhibit poor membrane fusion, cellular uptake and selective targeting capacities. To overcome these limitations, we used Amadori-glycated phosphatidylethanolamine, which is universally present in animals and commonly consumed in foods. We found that liposomes containing Amadori-glycated phosphatidylethanolamine exhibited significantly reduced negative membrane potential and demonstrated high cellular uptake.

## 1. Introduction

Liposomes are closed vesicles consisting of phospholipid bilayers that exhibit similarities to biological membranes. Owing to their high biocompatibility and ease of processing, liposomes have become a commercial reality that contribute to the daily lives of people, as they are found in drugs, cosmetics and processed foods [1,2]. In particular, liposomes are widely used as carriers in drug delivery systems (DDS) [3].

Well-known major routes of drug administration are intravenous (IV) and oral administration. However, following administration via both routes, a considerable amount of drug is delivered to unaffected cells/tissues (causing side effects) or quickly discharged from the body (resulting in increased healthcare expenditures), while only a small fraction of the drug is delivered to the target cells/tissues. Liposomes have the potential to improve these limitations, because they can control the retention of encapsulated drugs in circulation, sustained release, dispersibility, delivery to specific tissues and so on by modifying the chemical structure of the lipid formulation and its associated surfaces [3]. Therefore, liposomes have been extensively investigated as carriers in DDS and various applications in processed foods. Generally, liposomes are prepared from phosphatidylcholine (PC). However, liposomes consisting of 100% PC exhibit poor membrane fusion, cellular uptake and selective targeting capacities [4]. To overcome these limitations, efforts have focused on preparing liposomes from mixtures of PC and other phospholipids, such as phosphatidylethanolamine (PE). PE exhibits higher membrane fusion and cellular uptake capacities compared to PC [4,5]. Further, to increase the uptake of drugs into cells that highly express the glucose transporters (GLUTs) (e.g. cancer cells), liposomes containing saccharides that respond to GLUTs have been investigated as a means to enhance therapeutic effects [6–9].

**Figure 1.**
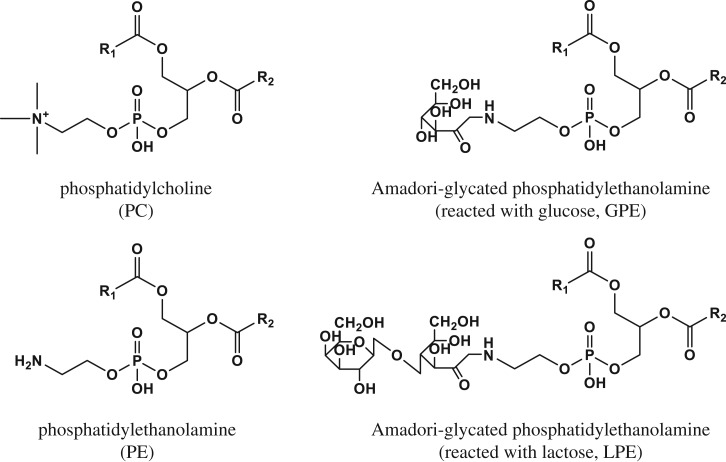
Chemical structures of lipids used in the present study. R1, R2 = palmitic acid.

Amadori-glycated PE (figure 1) is a Maillard reaction product that is universally present in animals. Also, glycated PE is commonly consumed as daily foods [10]. Unlike PE, glycated PE contains a saccharide moiety in its chemical structure. We previously reported a method to synthesize glycated PE standards with high purity from saccharides and PE [11,12]. We hypothesized that liposomes prepared from mixtures of PC and glycated PE should exhibit enhanced membrane fusion and cellular uptake capacities due to the presence of the PE sites, while the presence of the saccharide moiety should allow for selective targeting of cells that highly express GLUTs on their membranes.

As studies investigating liposomes containing glycated PE have not previously been reported, we herein aimed to examine the physical properties (particle size, membrane potential) of the liposomes, as well as their uptake into cells, to evaluate their potential for use in processed foods and as carriers in DDS.

To confirm the cellular uptake capacity of liposomes, we used the lipophilic polyphenol curcumin (CUR) as a model drug. CUR is a common food additive obtained from the plant turmeric (*Curcuma longa*), and known to have various physiological activities [13,14]. Moreover, CUR encapsulation in liposomes has previously been demonstrated [15,16].

## 2. Material and methods

### 2.1. Materials

l-α-Phosphatidylcholine dipalmitoyl (used as PC) and l-α-phosphatidylethanolamine dipalmitoyl (used as PE) were purchased from Wako Pure Chemical Industries (Osaka, Japan). Lipids were dissolved in chloroform (CHCl_3_) : methanol (MeOH) = 2 : 1 (v/v) at a concentration of 2 mg ml^−1^, and subsequently stored at −30°C. CUR was obtained from Wako Pure Chemical Industries, and was dissolved in CHCl_3 _: MeOH = 2 : 1 (v/v) at a concentration of 20 mg ml^−1^. β-Lactose (≤30% α-anomer basis, ≥99% total lactose basis) was purchased from Sigma-Aldrich (MO, USA). All other chemicals and reagents used were of analytical grade or higher.

### 2.2. Preparation of liposomes at different phospholipid ratios

Liposomes were prepared by the Bangham method [17]. Briefly, 20 mg of five different ratios of phospholipids (PC : PE = 1 : 0, 3 : 1, 1 : 1, 1 : 3 and 0 : 1) were dissolved in 10 ml of CHCl_3 _: MeOH = 2 : 1 (v/v) in eggplant flasks. Next, 200 µl of CUR solution (containing 4 mg CUR) was added to each flask. The solvent was then removed under vacuum on a rotary evaporator to prepare a thin lipid film. The resultant film was dissolved in 4 ml Milli-Q water and sonicated at 40% amplitude for 2 min at 1 s intervals with an ultrasonic homogenizer to obtain multilamellar vesicles (MLVs). The MLVs were passed through a polycarbonate membrane (PC Membranes 0.4 μm®, Avanti Polar Lipids, AL, USA) using a liposome extruder (Mini-Extruder®, Avanti Polar Lipids) to obtain liposomes. Non-encapsulated aggregates of CUR crystals were removed by filtration [18]. Ultrasonic treatment, filtering and liposomal preparation were carried out at the phase transition temperature [19].

### 2.3. Preparation of glycated phosphatidylethanolamine

**Figure 2.**
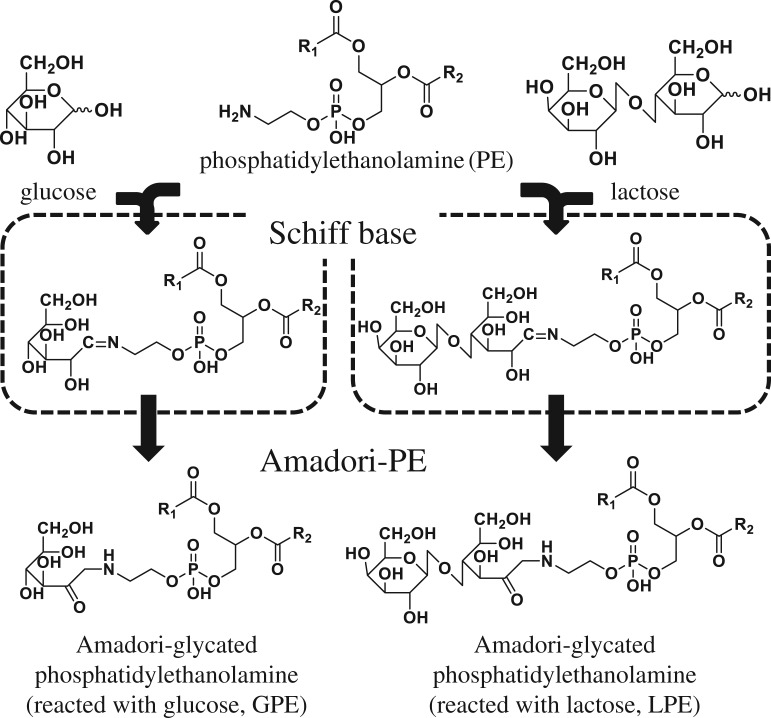
Scheme of the glycation of PE. The amino group of PE and the hydroxyl group of the saccharide (glucose and lactose) react via a dehydration condensation reaction to form an unstable ring-opening compound known as a Schiff base (–C = N–). This is followed by an Amadori rearrangement reaction that results in the glycated PE forming a ketone group at the C-2 position.

**Figure 3.**
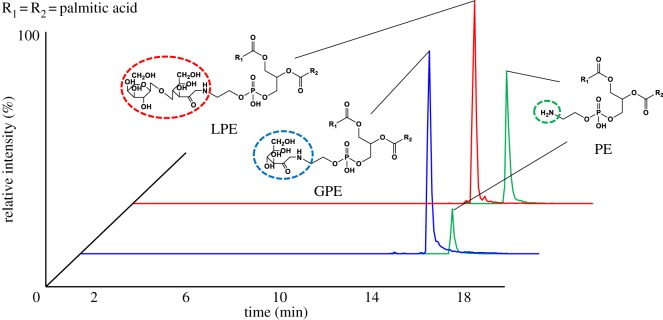
MRM chromatograms of glycated PE (GPE and LPE) and PE. GPE, LPE and PE were confirmed by HPLC coupled with tandem mass spectrometry (HPLC-MS/MS).

Glycated PE was prepared according to the method previously described by Miyazawa *et al*. [11,12]. In this glycation reaction, the amino group of PE and the hydroxyl group of the saccharide react via a dehydration condensation reaction to form an unstable ring-opening compound known as a Schiff base (–C = N–). This is followed by an Amadori rearrangement reaction that results in the glycated PE forming a ketone group at the C-2 position (figure 2). Briefly, PE was incubated with glucose, lactose or vehicle (55°C, 48 h). Reactants, glycated PE (glucose-PE (GPE) and lactose-PE (LPE)) and non-glycated PE were extracted by the Folch method (figure 1, PE, GPE and LPE) [20]. The production efficiency of glycated PE was confirmed by high-performance liquid chromatography (HPLC) coupled with tandem mass spectrometry (HPLC-MS/MS, 4000 QTRAP®, SCIEX, Tokyo, Japan) (figure 3). Specifically, an Atlantis T3 column (2.1 × 100 mm, 3 µm; Waters, MA, USA) was used, and the extract was eluted using a binary gradient consisting of the following HPLC solvents: A, water containing 5 mM ammonium acetate; B, MeOH containing 5 mM ammonium acetate. The gradient profile was as follows: B linear ratio; 70%–100% at 0–10 min, 100% at 10–15 min and 70% at 15–20 min. The flow rate was adjusted to 0.2 ml min^−1^, and the column temperature was maintained at 40°C. Multiple reaction monitoring (MRM) was carried out according to our previous report [21].

### 2.4. Preparation of liposomes consisting of phosphatidylcholine and glycated phosphatidylethanolamine (or control non-glycated phosphatidylethanolamine)

Liposomes were prepared from PC and glycated PE (or non-glycated PE) at a PC : glycated PE ratio of 3 : 1, according to the preparation scheme described above (§2.2).

### 2.5. Entrapment efficiency of curcumin in liposomes

Ten millilitres of the liposomal dispersion was mixed with 90 µl of MeOH to release CUR from the liposomes into the organic solvent. The amount of CUR released was determined by HPLC coupled with ultraviolet–visible detection (HPLC-UV). Specifically, a ProC18 column (4.6 × 150 mm, 5 µm; YMC, Kyoto, Japan) was used, and the extract was eluted using a binary gradient consisting of the following HPLC solvents: A, water containing 0.05% formic acid; B, acetonitrile. The gradient profile was as follows: B linear ratio; 30%–50% at 0–17 min, 50%–100% at 17–22 min and 100% at 22–32 min. The flow rate was adjusted to 1 ml min^−1^, and the column temperature was maintained at 40°C. The column eluent was directed to a UV detector (UV-2075 PLUS®, JASCO, Tokyo, Japan) for CUR monitoring at 420 nm. The concentration of CUR was calculated using the absolute calibration curve method. Encapsulation efficiency (EE) was calculated as follows: EE (%) = CURencapsulated/CURtotal × 100 [22].

### 2.6. Physical properties of liposomes

Based on the above results (§2.5), a 1 ml liposomal dispersion was prepared with an EE of 5%. The dispersion was subjected to dynamic light scattering (DLS) and laser Doppler anemometry measurements using an ELS-Z (Otsuka Electronics, Osaka, Japan) to evaluate the mean diameters and zeta potentials, respectively.

### 2.7. Culture of Caco-2 human epithelial colorectal adenocarcinoma cells

Caco-2 cells were cultured in Dulbecco's modified Eagle's medium (DMEM-5796; Sigma-Aldrich, MO, USA) containing 10% fetal bovine serum (FBS), 1% non-essential amino acid and 1% penicillin/streptomycin (hereinafter, the medium is referred to as ‘culture medium'). Caco-2 cells were grown on 10 cm plastic Petri dishes at 37°C in air and 5% CO_2_.

### 2.8. Evaluation of cellular uptake

Caco-2 cells with 80% confluence were treated with 0.05% trypsin–EDTA solution to detach the cells. After counting the number of cells in the cell suspension using a cell counting board (One Cell Counter®, Biomedical Sciences, Tokyo, Japan), cells were diluted with culture medium to yield a concentration of 4.0 × 10^5^ cells ml^−1^. Cells were seeded in 60 mm dishes (culture medium: 5 ml), and grown for 24 h in a CO_2_ incubator. After removing the medium and washing each dish with 5 ml of PBS (−), 5 ml of test medium (liposomes containing glycated PE or non-glycated PE dispersion in 1% FBS medium) equivalent to 100 μM CUR was added, and incubated for 24 h. After incubation, each dish was washed three times with 5 ml of PBS (−) and an additional 1 ml of a 0.05% trypsin–EDTA solution for cell detachment. Cells were completely detached by pipetting 4 ml of culture medium. The cell suspension was centrifuged (1000 g, 4°C, 5 min), and the cells were collected as the precipitate. Upon extraction of CUR from the recovered cells, 1 ml of PBS (−) was added and centrifuged again (1000 g, 4°C, 5 min) to wash the supernatant. Then, 100 µl of Milli-Q water was added to the cell precipitate, and the precipitate was disrupted by sonication. MeOH (200 µl) was added to exude CUR. The cell lysate was vortexed and centrifuged (5000 g, 4°C, 15 min). The CUR-containing supernatant was analysed by HPLC coupled with fluorescence detection (HPLC-FL) to evaluate cellular uptake of CUR-encapsulated liposomes. The conditions for HPLC were the same as described above (§2.5). The fluorescence detector was set to an excitation wavelength of 426 nm and an emission wavelength of 539 nm, based on the fluorescence properties of CUR [23].

### 2.9. Statistical analysis

Data were expressed as means ± standard deviation (s.d.). Statistical significance between groups was assessed using Dunnett's test with statistical software (Excel Statistics 2015®, SSRI, Tokyo, Japan). Means significantly differed at *p < 0.05 and **p < 0.01.

## 3. Results

**Figure 4.**
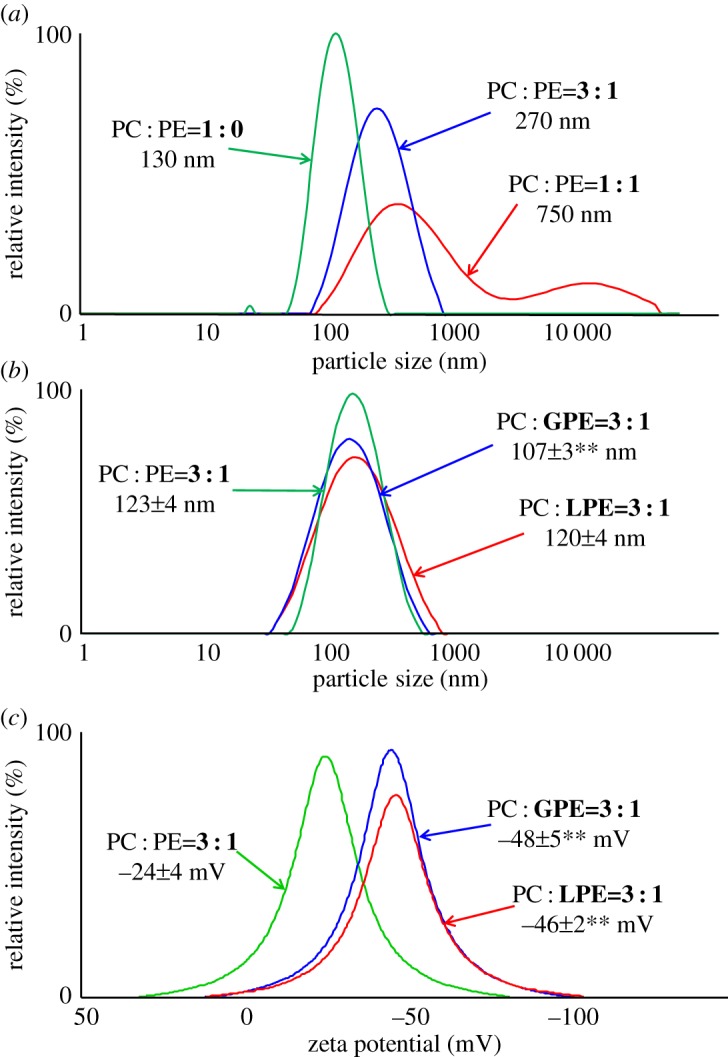
Average particle sizes and zeta potential distributions of liposomes. (*a*) Average particle size of liposomes prepared from different ratios of PC and PE; mean ± s.d. (*b*) Average particle size of liposomes prepared from different ratios of PC and glycated PE (GPE and LPE). (*c*) Zeta potential distribution of liposomes prepared from different ratios of GPE and LPE; mean ± s.d., *n* = 3. Means significantly differed at **p < 0.01.

**Figure 5.**
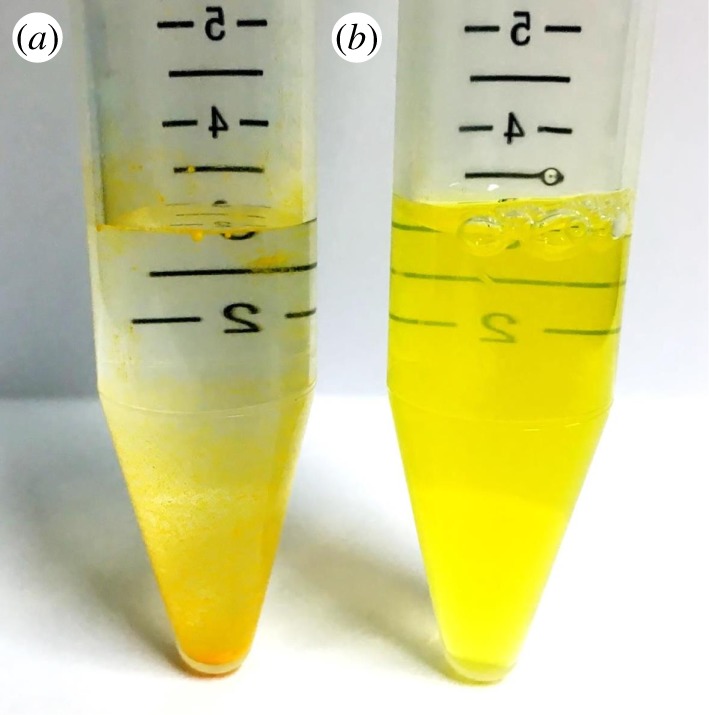
Photographs of suspensions of CUR and CUR-encapsulated liposomes. (*a*) CUR powder was re-dispersed in distilled water, mixed for 5 min, and then photographed; (*b*) liposomes (ratio of PC to GPE was adjusted to 3 : 1) were photographed after preparation.

**Figure 6.**
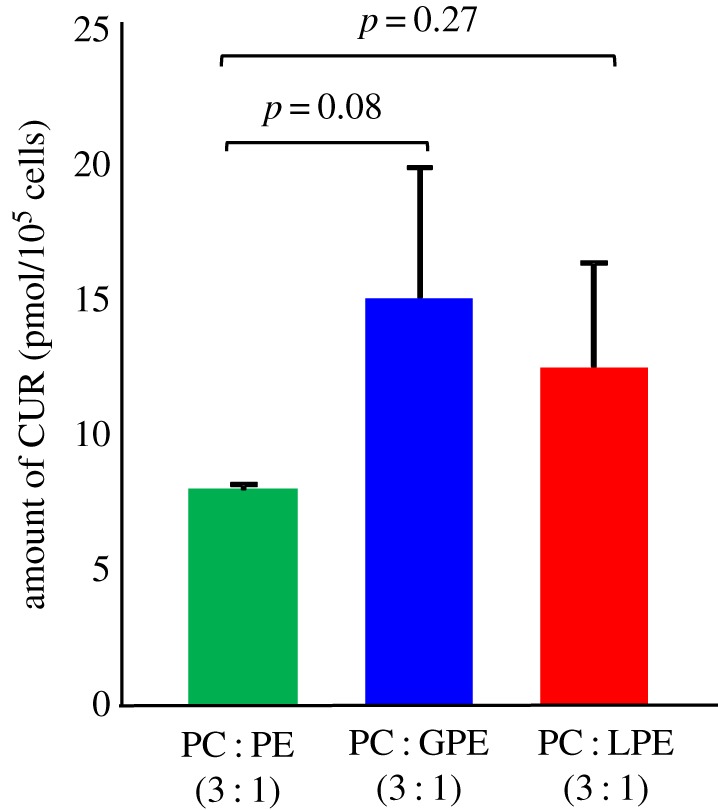
Cellular uptake of CUR-encapsulated small unilamellar vesicles. Small unilamellar vesicles were prepared from PC and glycated PE (GPE, LPE) or non-glycated PE. The ratio of PC to glycated PE (or non-glycated PE) was adjusted to 3 : 1 in all groups. Statistical significance between groups was assessed by Dunnett's test. Data are mean ± s.d., *n* = 3.

In this study, we first determined a suitable compositional ratio of PC and PE for the preparation of liposomes. We then prepared liposomes containing glycated PE and evaluated their physical properties. We also evaluated the cellular uptake capacity of CUR-encapsulated liposomes containing glycated PE into Caco-2 human epithelial colorectal adenocarcinoma cells, which are known to highly express GLUTs [24,25]. Among the five phospholipid ratios evaluated (PC : PE = 1 : 0, 3 : 1, 1 : 1, 1 : 3 and 0 : 1) for liposome preparation, precipitation of unencapsulated CUR crystals was observed after sonication using PC : PE ratios of 1 : 3 and 0 : 1 [18]. Owing to accumulation of aggregates in the dispersion, it was difficult to prepare liposomes using these ratios. On the other hand, at PC : PE ratios of 1 : 0, 3 : 1 and 1 : 1, a homogeneous MLV dispersion was obtained by sonication after the addition of Milli-Q water. Mean diameters of liposomes prepared at PC : PE ratios of 1 : 0, 3 : 1 and 1 : 1 were 130, 270 and 750 nm, respectively, as measured by DLS. Mean diameters increased with an increase in the PE ratio (figure 4*a*). The unimodal distribution obtained by DLS suggests that liposomes prepared at PC : PE ratios of 1 : 0 and 3 : 1 were homogeneously dispersed in solution. On the other hand, liposomes prepared at a PC : PE ratio of 1 : 1 did show a bimodal distribution. For the synthesis of glycated PE (both GPE and LPE), more than half of PE converted to glycated PE in the synthesis (determined by HPLC-MS/MS). The mean diameters of liposomes containing non-glycated PE or glycated PE were around 100 nm, as measured by DLS (figure 4*b*). On the other hand, the zeta potentials of liposomes containing glycated PE were significantly lower than those containing non-glycated PE (figure 4*c*). For all liposomes, the EE of CUR was around 10%, and no differences in appearance of the liposomal dispersions were noted (figure 5). The cellular uptake of CUR was analysed using HPLC-FL. After incubation for 24 h, liposomes containing glycated PE tended to increase the uptake of CUR into Caco-2 cells, relative to liposomes containing non-glycated PE (figure 6).

## 4. Discussion

In this study, we developed liposomes containing glycated PE. Then, we searched the possibility of application as carriers in DDS and use in processed foods. With regard to the former, we measured the physical properties and cellular uptake capacities of the prepared liposomes. We first examined the ratio of PC and PE for liposome preparation. Liposomes prepared at PC : PE ratios of 1 : 0 and 3 : 1 showed unimodal distributions, as measured by DLS (figure 4*a*), suggesting that liposomes prepared at these ratios were uniformly dispersed in the system. On the other hand, liposomes prepared at a PC : PE ratio of 1 : 1 show a bimodal distribution, and the mean diameter was larger (750 nm) than those prepared at other ratios (PC : PE = 1 : 0, 130 nm; 3 : 1, 270 nm). Therefore, a PC : PE ratio of 1 : 1 was considered to be unsuitable for preparation of liposomes. In addition, we also took into account that liposomes could not be prepared if the ratio of PE to PC was greater than one-third. It is well known that assemblies prepared from PC form a cylindrical shape, while assemblies prepared from PE form a conical shape [1]. At a PE : PC ratio greater than one-third, the lipid bilayer of the resulting liposomes is expected to be unstable due to the shape differences between PC and PE. Therefore, we found that the ratio of PE to PC needs to be less than or equal to one-third to prepare liposomes with a uniform particle size distribution.

The EE of CUR in liposomes was around 10%. This value is consistent with that previously obtained for CUR-encapsulated liposomes formed from 100% PC (EE of around 8%) [26]. In addition, it was reported that CUR molecules in the liposomes are localized and stabilized between the fatty acid acyl groups [27]. In the current study, liposomes were prepared with the same substrates as used previously, and their sizes showed a unimodal distribution. Based on these results, the CUR molecules in this study were suggested to be localized at the fatty acid acyl groups of the liposomes and uniformly dispersed in solution. The synthesis efficiency of glycated PE was determined by HPLC-MS/MS to be greater than 50% for both GPE and LPE. These results are consistent with previous studies conducted in our laboratory [11,12]. On the other hand, liposomes containing glycated PE were found to exhibit a significant reduction in negative zeta potential, compared with liposomes containing non-glycated PE (figure 4*c*). This reduction may be attributable to the hydrophilic sugar moiety of glycated PE [28]. The zeta potential is an indicator of the stability of liposomal dispersions. Generally, as the absolute value of the surface charge increases, repulsion between liposomes increases and aggregation is minimized. Therefore, it is thought that liposomes consisting of glycated PE in a dispersion are more stable than liposomes consisting of non-glycated PE. Further, as the surfaces of animal cells exhibit a negative charge [29], substances with weak negative and positive charges adhere nonspecifically to the cells. Accordingly, control of these substances in animals is known to be greatly limited. On the other hand, liposomes containing glycated PE prepared in this study exhibited a negative surface charge, suggesting that they should exhibit less nonspecific adsorption *in vivo* than liposomes containing 100% PC. Thus, liposomes containing glycated PE may be useful as drug carriers in DDS for the purpose of controlling retention in the blood and subsequent delivery to specific cells/tissues. On the other hand, we did not confirm the time-dependent stability of the liposomes. More research is needed to clarify such details.

**Figure 7.**
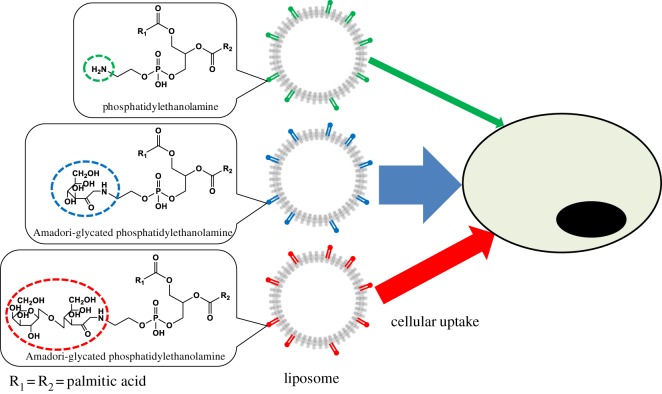
Assumed behaviour of liposomes used in the present study.

Evaluation of the cellular uptake of liposomes prepared from PC and glycated PE (or non-glycated PE) demonstrated that liposomes containing glycated PE tended to increase the uptake of CUR into Caco-2 cells compared with liposomes containing non-glycated PE (figure 6). Generally, actively dividing cell types such as cancer cells express GLUTs at high levels because they require large amounts of sugar as nutrients [6]. In addition, GLUTs are highly expressed in normal cells of the human small intestine, and are known to play a key role in the digestion and absorption of sugars [25]. GLUTs are also highly expressed in Caco-2 cells, which are human colon cancer-derived cells. Therefore, it can be inferred that liposomes containing glycated PE are taken up by Caco-2 cells due to the presence of the saccharide moiety in glycated PE, which binds to GLUTs. On the other hand, levels of cellular uptake of liposomes containing GPE tended to be higher than those of liposomes containing LPE (GPE: *p* = 0.08, LPE: *p* = 0.27). GLUTs are known to recognize the structure of monosaccharides at the time of sugar uptake into cells [30]. Therefore, it is speculated that GPE, which contains the monosaccharide glucose, was more easily recognized by GLUTs, which resulted in the increased cellular uptake compared with LPE, which contains the disaccharide lactose (figure 7). This phenomenon can be used to enhance the selective targeting and subsequent cellular uptake of liposomes via not only IV but also oral administration.

## 5. Conclusion

From this study, it is considered that liposomes containing glycated PE are more stable than liposomes containing non-glycated PE, and are taken up by cells that highly express GLUTs. These characteristics should allow for various potential applications, such as increased uptake of encapsulated drugs to target cells following IV injection, and enhancement of absorption of encapsulated nutrients in the intestine. Future studies are needed to investigate these possibilities. Taken together, the results of this study suggest that glycated PE may enhance the delivery of drugs and nutritional components.

## Data accessibility

The datasets supporting this article can be found at https://dx.doi.org/10.5061/dryad.g6f6g [31].
